# RNA-dependent protein kinase (PKR) depletes nutrients, inducing phosphorylation of AMP-activated kinase in lung cancer

**DOI:** 10.18632/oncotarget.3573

**Published:** 2015-03-14

**Authors:** Chengcheng Guo, Chuncheng Hao, RuPing Shao, Bingliang Fang, Arlene M. Correa, Wayne L. Hofstetter, Jack A. Roth, Carmen Behrens, Neda Kalhor, Ignacio I. Wistuba, Stephen G. Swisher, Apar Pataer

**Affiliations:** ^1^ Departments of Thoracic and Cardiovascular Surgery, The University of Texas MD Anderson Cancer Center, Houston, TX, USA; ^2^ Thoracic Head and Neck Medical Oncology, The University of Texas MD Anderson Cancer Center, Houston, TX, USA; ^3^ Pathology, The University of Texas MD Anderson Cancer Center, Houston, TX, USA; ^4^ Translational Molecular Pathology, The University of Texas MD Anderson Cancer Center, Houston, TX, USA; ^5^ Current address: Sun Yat-Sen University Cancer Center, State Key Laboratory of Oncology in South China, Guangzhou, China; ^6^ Current address: Department of Oncology Radiotherapy, The Cancer Hospital of Harbin Medical University, Harbin, Heilongjiang, People's Republic of China

**Keywords:** PKR, AMPK, nutrient depletion, lung cancer

## Abstract

We have demonstrated that RNA-dependent protein kinase (PKR) and its downstream protein p-eIF2α are independent prognostic markers for overall survival in lung cancer. In the current study, we further investigate the interaction between PKR and AMPK in lung tumor tissue and cancer cell lines. We examined PKR protein expression in 55 frozen primary lung tumor tissues by Western blotting and analyzed the association between PKR expression and expresson of 139 proteins on tissue samples examined previously by Reverse Phase Protein Array (RPPA) from the same 55 patients. We observed that biomarkers were either positively (phosphorylated AMP-activated kinase^T172^ [p-AMPK]) or negatively (insulin receptor substrate 1, meiotic recombination 11, ATR interacting protein, telomerase, checkpoint kinase 1, and cyclin E1) correlated with PKR. We further confirmed that induction of PKR with expression vectors in lung cancer cells causes activation of the AMPK protein independent of the LKB1, TAK1, and CaMKKβ pathway. We found that PKR causes nutrient depletion, which increases AMP levels and decreases ATP levels, causing AMPK phosphorylation. We further demonstrated that inhibiting AMPK expression with compound C or siRNA enhanced PKR-mediated cell death. We next explored the combination of PKR and p-AMPK expression in NSCLC patients and observed that expression of p-AMPK predicted a poor outcome for adenocarcinoma patients with high PKR expression and a better prognosis for those with low PKR expression. These findings were consistent with our *in vitro* results. AMPK might rescue cells facing metabolic stresses, such as ATP depletion caused by PKR. Our data indicate that PKR causes nutrient depletion, which induces the phosphorylation of AMPK. AMPK might act as a protective response to metabolic stresses, such as nutrient deprivation.

## INTRODUCTION

RNA-dependent protein kinase (PKR) has a well-established role in antiviral defense mechanisms as well as in other cellular functions, such as growth control, apoptosis regulation, cell proliferation, signal transduction, and differentiation [1-4]. In many types of cancer cells, overexpression or activation of PKR leads to apoptosis, which may result from increased phosphorylation of eukaryotic initiation factor 2 alpha (eIF2α) [2,4]. Studies have shown that increased expression of PKR correlates with a better prognosis in cancers of the head, neck, and colon [4,5]. Our laboratory has shown that the PKR pathway is necessary for inducing cell death in many types of cancer cells after various treatments [6-9]. We also previously demonstrated that PKR and its downstream protein, phosphorylated eIF2α (p-eIF2α), are independent prognostic markers for overall survival in patients with non-small cell lung cancer (NSCLC) [10, 11].

We previously determined the differential expression of proteins in primary lung tumor tissues and normal lung tissues from the same patients by using a reverse-phase protein array (RPPA) assay. To further use existing RPPA data to determine PKR network, we examined PKR expression again using left over protein samples from RPPA assay by Western blot analysis. We used Western blotting to examine PKR protein expression in 55 available frozen primary lung tumor tissues and analyzed the association between expression of PKR and expression of 139 proteins in frozen primary lung tumor tissues from the same 55 patients in our earlier RPPA analysis [12]. We found biomarkers that were either positively (phosphorylated AMP-activated protein kinase^T172^[p-AMPK]) or negatively (insulin receptor substrate 1 [IRS1], meiotic recombination 11 [MRE11], ATR interacting protein [ATRIP], telomerase, checkpoint kinase 1 [CHK1], and cyclin E1) correlated with PKR. AMPK, a serine/threonine (Thr) protein kinase, is activated by cellular stresses that deplete ATP and is a key factor in cancer metabolism [13-15]. AMPK responds to increases in the AMP/ATP ratio by switching off ATP-consuming pathways, such as fatty acid synthesis or gluconeogenesis, and by switching on pathways for ATP generation, such as glycolysis and amino acid oxidation [13,14]. AMPK is activated by phosphorylation of the catalytic subunits at Thr172, which is mediated by liver kinase B1 (LKB1), transforming growth factor beta-activated kinase 1 (TAK1), and calmodulin-dependent protein kinase kinase (CaMKKβ) [13, 14]. AMPK regulates energy homeostasis in mammalian cells through multiple pathways, including inhibiting lipid synthesis by acetyl-CoA carboxylase-1 (ACC1) phosphorylation and inhibiting mammalian target of rapamycin (mTOR)-dependent protein translation through regulating the tuberous sclerosis complex 1 or 2 (TSC1/2) [15,16]. Studies have shown that p-AMPK is a prognostic marker and that high p-AMPK expression levels are associated with increased overall survival in patients with lung cancer and gastric cancer [17,18]. Therefore, we focused on p-AMPK and hypothesized that inducing PKR with expression vectors in lung cancer cells activates AMPK and that the PKR-AMPK pathway may play an important role on prognosis of lung cancer.

In the present study, we demonstrated that inducing PKR in lung cancer cells activates the AMPK protein independent of the LKB1, TAK1, and CaMKKβ pathways by increasing AMP levels and decreasing ATP levels. We observed that inhibiting AMPK expression by compound C or AMPK siRNA enhanced PKR-mediated cell death. We further explored the combination of PKR and p-AMPK expression in NSCLC patients and observed that p-AMPK promotes cancer cell survival in adenocarcinoma patients with high PKR expression and inhibits cancer cell growth in adenocarcinoma patients with low PKR levels. Our data indicate that PKR causes nutrient depletion, which induces phosphorylation of AMPK, a process that is required for rescue cells from metabolic stresses.

## RESULTS

### Association between PKR and AMPK in lung tumors

**Figure 1:**
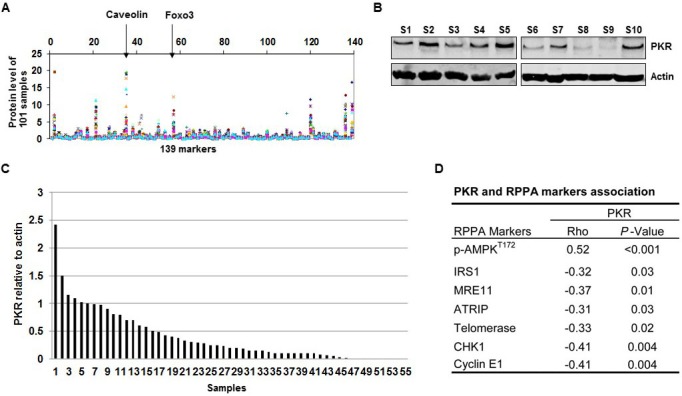
RNA-dependent protein kinase (PKR) levels are correlated with AMP-activated kinase (AMPK) protein levels in primary non-small cell lung cancer tissues (A) Levels of 139 protein markers, including caveolin and forkhead box O3 (Foxo3), on 101 frozen primary lung tumor samples were determined by RPPA assay. (B) Western blotting of PKR protein expression in 10 (S1-S10, Sample 1-10) of 55 human lung tumor samples. The expression of actin was used as a loading control. (C) Densitometric analysis results of the ratio of PKR to β-actin show normalized protein levels for the 55 tumor samples. (D) PKR protein expression was positively correlated with phosphorylated (p-)AMPK (Spearman's rho= 0.52, *P* < 0.001) and was negatively correlated with several markers (insulin receptor substrate 1 [IRS1], meiotic recombination 11 [MRE11], ATR interacting protein [ATRIP], telomerase, checkpoint kinase 1 [CHK1], and cyclin E1).

In an earlier RPPA study, we examined 101 frozen tissue lysates from NSCLC patients for expression of 139 protein markers. In the current study, due to limited availability of samples, we were only able to analyze 55 of the 101 frozen tissue lysates for PKR protein expression by Western blotting. RPPA results showed different levels of 139 proteins, including caveolin and forkhead box O3, among the 101 samples (Figure 1A). Figure 1B shows 10 of these 55 frozen samples examined by Western blotting (Figure 1B). Western blotting results showed that 55 tumor samples expressed different levels of PKR at the protein levels (Figure 1C). We next determined the association between PKR and those 139 biomarkers and found biomarkers that were positively (p-AMPK) and negatively (IRS1, MRE11, ATRIP, telomerase, CHK1, and cyclin E1) correlated with PKR (Figure 1D).

### PKR mediates AMPK activation

**Figure 2:**
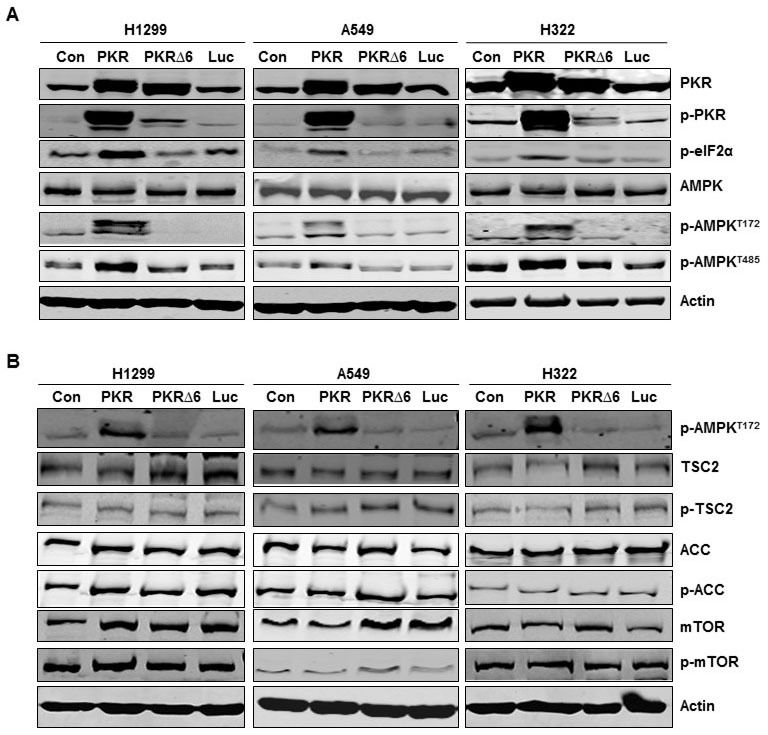
Inducing RNA-dependent protein kinase (PKR) mediates AMP-activated kinase (AMPK) activation in non-small cell lung cancer cells (A) and (B) Western blotting of PKR, phosphorylated (p-)PKR, p-eIF2α, AMPK, p-AMPK^T172^, p-AMPK^T485^, tuberous sclerosis complex 2 (TSC2), p-TSC2, acetyl-CoA carboxylase (ACC), p-ACC, mammalian target of rapamycin (mTOR), and p-mTOR protein expression in human lung cancer cell lines (H1299, A549, and H322) 72 hours after transfection with PBS (control [con]), Ad-PKR (2,500 viral particles/cell), Ad-PKRΔ6 (2,500 viral particles/cell), or Ad-Luc (2,500 viral particles/cell). The expression of actin was used as a loading control.

To further confirm the positive correlation between PKR and p-AMPK, we determined whether induction of PKR with expression vectors in H1299, A549, and H322 lung cancer cells activates AMPK protein. H1299, A549, and H322 cancer cells transfected with Ad-PKR (wild type PKR) or Ad-PKRΔ6 (mutant PKR) had comparable expression of PKR protein, but only cells transfected with Ad-PKR had increased expression of phosphorylated PKR at Thr451 (p-PKR) and phosphorylated eIF2α at Ser51 (p-eIF2α). We did not detect increased expression of p-PKR or p-eIF2α in cancer cells transfected with Ad-PKRΔ6 or Ad-Luc (control vector) (Figure 2A). Of compared cells transfected with control, mutant PKR (PKRΔ6), or Luc, only wild-type PKR-transfected cells showed activation of AMPK, with increased phosphorylation of AMPKα at Thr172 (Figure 2A). We also observed that PKR transfection sometimes induced immunoreactivity at two adjacent bands of lower mobility (Figure 2A). Additional structural or conformational changes associated with AMPK activation must be responsible for this mobility shift. Phosphorylation at additional sites is quite probable, particularly since Ser485 is in the AMPK a-subunit. Compared with the control, mutant PKR, or Luc transfection, wild-type PKR transfection showed activated AMPK, with increased phosphorylation of AMPKα at Thr485 (Figure 2A). To assess whether the activation of AMPK by PKR was due to direct interaction with AMPK or to the induction of other protein intermediates, we performed coimmunoprecipitation studies with PKR and AMPK. We did not detect direct interaction between PKR and AMPK (data not shown). AMPK activation regulates many substrates, such as TSC2, ACC, and mTOR, which all have an essential role in regulating cell growth and proliferation. We next used Western blotting to determine how the phosphorylation of ACC, TSC2, and mTOR correlate with the phosphorylation of AMPK after transfection with vectors (wild-type PKR, mutant PKR, and Luc) in H1299, A549, and H322 lung cancer cells. We did not detect any expression change of TSC2, ACC, and mTOR phosphorylation in these cancer cells after induction of PKR (Figure 2B).

### PKR activates AMPK activation by inhibiting ATP

**Figure 3:**
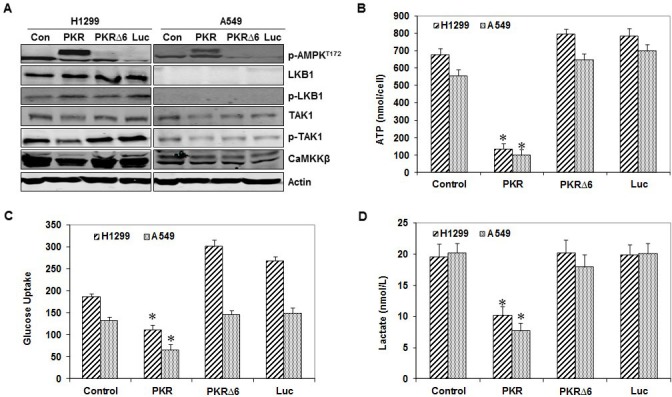
RNA-dependent protein kinase (PKR) mediates AMP-activated kinase (AMPK) activation by inhibiting ATP (A) Western blotting of phosphorylated (p-)AMPK, liver kinase B1 (LKB1), p-LKB1, transforming growth factor-b-activated kinase-1 (TAK1), p-TAK1, and calmodulin-dependent protein kinase kinase (CaMKKβ) protein expression in human lung cancer cell lines (H1299 and A549) 72 hours after transfection with PBS (control [con]), Ad-PKR (2,500 viral particles/cell), Ad-PKRΔ6 (2,500 viral particles/cell), or Ad-Luc (2,500 viral particles/cell). The expression of actin was used as a loading control. The levels of ATP (B), glucose uptake (C), and lactate production (D) were measured in human lung cancer cell lines (H1299 and A549) 72 hours after transfection with PBS (control), Ad-PKR (2,500 viral particles/cell), Ad-PKRΔ6 (2,500 viral particles/cell), or Ad-Luc (2,500 viral particles/cell). Compared with the PBS control (con), Ad-PKRΔ6, or Ad-Luc, Ad-PKR decreased cellular ATP levels (B), inhibited the cell glucose uptake (C), and decreased lactate production (D). Experiments were performed in triplicate; data represent the mean (SD). *P* values less than 0.05 indicated by asterisks.

LKB1, TAK1, and CaMKKβ are the predominant kinases upstream of AMPK [13,14]. Previous studies have shown that LKB1 protein expression was absent in A549 cells but present in H1299 cells [19]. Inducing PKR caused phosphorylation of AMPK in A549 cells, suggesting that PKR activates AMPK independently of LKB1 (Figure 3A). AMPK activation in these cancer cells was not likely due to increases in CaMKKβ levels because inducing PKR reduced CaMKKβ levels in H1299 and A549 cells (Figure 3A). We next examined the effect of PKR on TAK1 activation by assessing TAK1 phosphorylation at Thr184/187, an essential step for complete TAK1 activation. We did not detect enhancement of TAK1 phosphorylation in H1299 or A549 cells after inducing PKR (Figure 3A). Taken together, our results indicate that PKR activates AMPK in lung cancer cells independently of LKB1, TAK1, and CaMKKβAMPK. AMPK is also activated by increased AMP levels and decreased ATP levels. Studies have shown that AMP protects against the dephosphorylation of Thr172 and is another major physiological mechanism for activating AMPK. We next determined whether increased AMPK phosphorylation is due to increases in intracellular AMP levels in wild-type PKR-transfected H1299 and A549 lung cancer cells. Compared with control, mutant PKR, or Luc, only wild-type PKR transfection showed reduced intracellular ATP, glucose uptake, and lactate production (Figure 3B, 3C, and 3D). These results indicate that PKR activates AMPK in lung cancer cells by decreasing ATP levels and increasing AMP levels.

### Inhibiting AMPK enhances PKR-mediated cell death

**Figure 4:**
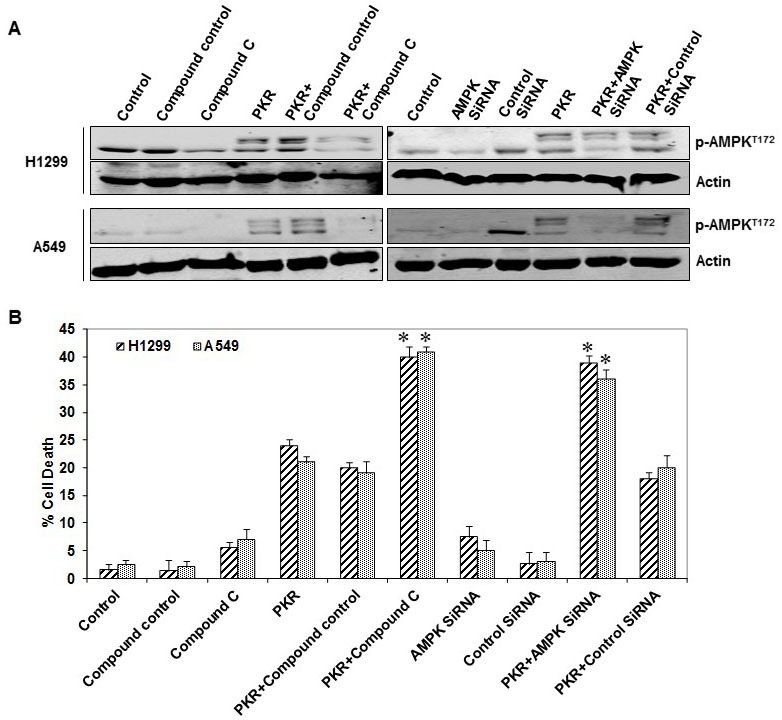
AMP-activated kinase (AMPK) inhibition enhances RNA-dependent protein kinase (PKR)-mediated cell death (A) Western blotting of phosphorylated (p-)AMPK protein expression in human lung cancer cell lines (H1299 and A549) 72 hours after transfection with Ad-PKR (2,500 viral particles/cell) with or without compound C, AMPK siRNA, compound control, or siRNA control. The expression of actin was used as a loading control. (B) Flow cytometric analysis of cell death in human lung cancer cell lines (H1299 and A549) 72 hours after transfection with Ad-PKR (2,500 viral particles/cell) with or without compound C, AMPK siRNA, compound control or siRNA control. Experiments were performed in triplicate; data represent the mean (SD). *P* values less than 0.05 indicated by asterisks.

Next, we investigated whether inhibiting AMPK affected PKR-mediated cell death. We examined the effects of Ad-PKR, alone and in combination with compound C or AMPK siRNA, in H1299 and A549 lung cancer cell lines. We demonstrated that PKR-mediated AMPK activation was blocked by compound C or AMPK siRNA but not by the control compound or control siRNA (Figure 4A). Flow cytometric analysis showed that transfection with only Ad-PKR resulted in cell death rates of 24% in H1299 cells and of 21% in A549 cells. Compound C treatments resulted in cell death rates of 5.5% in H1299 cells and of 7% in A549 cells after 72 hours. The combination of Ad-PKR and compound C resulted in substantially enhanced apoptosis in both H1299 (40%) and A549 (41%) lung cancer cells (Figure 4B). AMPK siRNA treatment resulted in cell death rates of 7.5% in H1299 cells and of 5% in A549 cells after 72 hours. The combination of Ad-PKR and AMPK siRNA substantially enhanced apoptosis in both H1299 (39%) and A549 (36%) lung cancer cells (Figure 4B). Apoptotic effects were not enhanced in cells treated with a combination of control compound or control siRNA (Figure 4B). Thus, our results suggest that AMPK may block apoptosis in PKR-transfected cancer cells, promoting cancer cell survival.

### Prognostic value of PKR and AMPK in NSCLC patients

**Figure 5:**
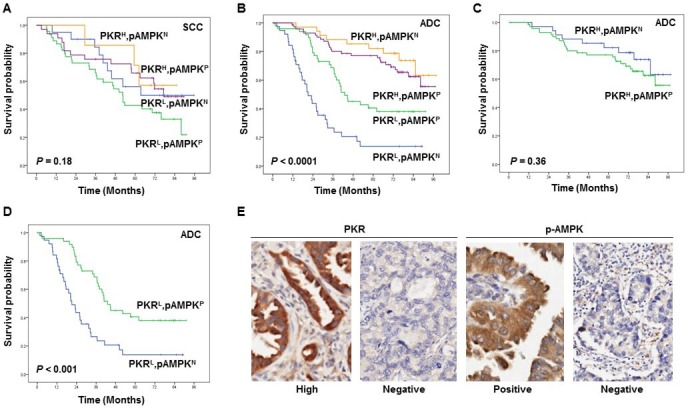
The prognostic significance assessed with Kaplan-Meier survival estimates and log-rank test (A-E) Kaplan-Meier survival curves showing the differences in survival duration using PKR combined with phosphorylated (p-)AMPK in all stages of squamous cell carcinoma (SCC) (A) and adenocarcinoma (ADC) (B-D) lung cancer patients. The survival rate in ADC patients with PKR^low^/p-AMPK^negative^ was significantly lower than that in ADC patients with PKR^low^/p-AMPK^positive^, ADC patients with PKR^high^/p-AMPK^positive^, and ADC patients with PKR^high^/p-AMPK^negative^ (B-D). Immunohistochemical staining examples for the expressions of PKR and p-AMPK in the cytoplasm of NSCLC cells (original magnification x400) (E).

We next analyzed the effects of the combination of PKR and p-AMPK on the overall survival of 299 NSCLC patients (194 adenocarcinoma and 105 squamous cell carcinoma). We used PKR and p-AMPK levels to stratify patients into four groups: those with high expression of PKR and with p-AMPK expression; those with high PKR expression and without p-AMPK expression; those with low PKR expression and with p-AMPK expression; and those with low PKR expression and without p-AMPK expression. Among the four stratified groups, overall survival did not differ significantly for squamous cell carcinoma patients (P = 0.18; Figure 5A); however, adenocarcinoma patients did have significantly different overall survival (P < 0.0001, Figure 5B). Among 194 adenocarcinoma patients, 72 PKR^High^/p-AMPK^Positive^ patients had slightly worse overall survival than did 34 PKR^High^/p-AMPK^Negative^ patients (Figure 5B and 5C). In addition, we observed that 50 PKR^Low^/p-AMPK^Positive^ patients had better overall survival than did 38 PKR^Low^/p-AMPKN^egative^ patients (Figure 5B and 5D). Representative images of PKR and p-AMPK expression in the cytoplasm of NSCLC cells are shown in Figure 5E. We also determined the PKR-p-AMPK relationship using Fisher's exact test and Spearman's correlation coefficient. We have demonstrated that PKR is positively correlated with p-AMPK in patients' samples (Spearman's rho=0.12; P = 0.03). Univariate and multivariate Cox proportional hazards regression analysis revealed that pathologic stage and PKR/p-AMPK expression significantly affected overall survival (Data not shown). Our results suggest that p-AMPK may promote tumor growth in adenocarcinoma patients with high PKR expression and may suppress tumor growth in adenocarcinoma patients with low PKR expression.

## DISCUSSION

We and others have previously demonstrated that increased expression of PKR correlates with a better prognosis in leukemia; in cancers of the lung, colon, and head and neck; and in other hematopoietic malignancies [4,10, 20]. In the present study, we demonstrated that only p-AMPK positively correlated with PKR in 55 frozen primary lung tumor tissues. Studies have reported that p-AMPK is a prognostic marker and that high p-AMPK expression levels are associated with increased survival in patients with gastric and lung cancer [17,18]. Therefore, we focused on AMPK and confirmed that only wild-type PKR transfection showed activated AMPK, with increased phosphorylation of AMPKα at Thr172 and Thr485 in lung cancer cells. We observed that PKR transfection sometimes induced two adjacent bands of lower mobility. AICAR (AMPK activator) as well as toxin agents (okadaic acid, microcystin-LR, calyculin A, cantharidin, and tautomycin) can induce low-mobility forms of AMPK alpha [21]. The low-mobility forms of AMPK could result from additional structural or conformational changes associated with AMPK activation or phosphorylation at additional sites. Further investigation is needed to determine whether PKR transfection increases the susceptibility of AMPK to phosphorylation at several sites.

AMPK activation plays an essential role in the PKR-mediated inhibition of mTOR signaling and the activation of TSC2 and ACC [13,14]. We expected that phosphorylation of TSC2 or ACC would correlate with p-AMPK after treatment with wild-type PKR in H1299, A549, and H322 cancer cells. To our surprise, we did not detect any change in the phosphorylation of TSC2, ACC, or mTOR in H1299, A549, and H322 cancer cells after inducing PKR. These results suggest that structural or conformational changes associated with AMPK phosphorylation at additional sites may impair some substrates of p-AMPK. These results also suggest that p-AMPK may regulate different pathways in these cancer cells. Currently, we are unable to explore other AMPK phosphorylation sites, owing to the limited availability of p-AMPK antibodies.

ATP-consuming processes such as glucose deprivation or hypoxia cause AMPK activation either via an activating upstream kinase (LKB1, TAK1, and CaMKKβ) or via changes within the intracellular ratio of AMP to ATP [13,14]. Our data indicated that AMPK phosphorylation was caused by increased AMP levels and decreased ATP levels, both of which were due to PKR-mediated nutrient depletion; the phosphorylation of AMPK was independent of the LKB1, TAK1, and CaMKKβ pathway. In cancer cells, phosphorylation of AMPK has been positively correlated with increases in glucose uptake [13-15, 22]. We demonstrated that the induction of PKR was responsible for nutrient deprivation, including decreasing ATP, inhibiting glucose uptake, and decreasing lactate concentration. The inhibition of glycolysis by PKR had indirect effects on various signaling pathways. We believe that PKR-induced AMPK activation was a consequence of glycolysis inhibition. The inhibition of glycolysis by PKR led to a decreased intracellular ATP concentration but an increased intracellular AMP concentration. AMP can bind to AMPK and alter AMPK conformation, resulting in AMPK activation.

AMPK monitors and maintains energy homeostasis at the cellular level; because AMPK primarily acts as a component of the LKB1 tumor suppressor cascade upstream of the TSC1/2/mTOR pathway, AMPK is likely to be a tumor suppressor [13,14]. However, AMPK has recently been shown to promote cancer cell survival in response to extrinsic and intrinsic stressors, including bioenergetic, growth factor, and oncogene stress [16,22, 23]. To address how AMPK activation affects cancer cell survival, we tested the p-AMPK level and cell death in PKR-treated cancer cells after treatment with AMPK siRNA and compound C (AMPK inhibitor). We demonstrated that inhibiting p-AMPK expression with compound C or siRNA enhanced PKR-mediated cell death in lung cancer cells. AMPK activation is essential for metabolic adaptation and cell survival during acute nutrient deprivation by blocking translation elongation [23]. Our data support this finding and suggest that PKR-mediated nutrient depletion induces phosphorylation of AMPK, which is required for cancer cell survival. We further explored the combination of PKR and p-AMPK expression in cells from NSCLC patients and observed that expression of p-AMPK predicts a worse prognosis in adenocarcinoma patients with high PKR levels, whereas expression of p-AMPK predicts a better prognosis in adenocarcinoma patients with low levels of PKR. Consistent with our *in vitro* results in primary lung tumors, p-AMPK may be required to rescue cells from metabolic stresses due to PKR-induced nutrient depletion.

We also found several biomarkers that were negatively correlated with PKR: IRS1, MRE11, ATRIP, telomerase, CHK1, and cyclin E1. IRS1 is an adaptor protein that is important for regulating proliferation, metabolism, and differentiation and that is a key mediator of insulin receptor and insulin-like growth factor-1 receptor functions [24]. MRE11 (meiotic recombination 11) and ATRIP (ATR interacting protein) play an important role in maintaining telomeres and response to replication stress during S-phase [25-27]. CHK1 is a principal regulator of the cell cycle that controls the initiation of DNA replication, stabilizes replication forks, and coordinates mitosis [28]. Cyclin E also plays a direct role in the initiation of DNA replication and in the control of genomic stability and has been associated with the initiation or progression of various human cancers [29,30]. Further studies are needed to confirm the negative correlation of PKR with these markers *in vitro* and to determine whether PKR is involved in DNA replication since most of these proteins are involved in this process.

In conclusion, AMPK has both tumor-suppressing and metabolic stress response capabilities. AMPK's role in tumor growth could depend on the mechanism of AMPK activation, on signaling networks, and on extracellular environmental conditions. In the current study, our data from lung cancer cells and human lung tumors suggest that there is crosstalk among PKR, AMPK, and nutrient depletion. PKR activated AMPK with increased phosphorylation of AMPKα. This phosphorylation of AMPKα appeared to be enzymatically inactive in relation to TSC2, ACC, and mTOR phosphorylation but still seemed able to promote cancer cell survival. AMPK may play a tumor-suppressing role in the absence of PKR and may play a rescue role in the presence of PKR. Because AMPK has been considered a viable target for cancer treatment in the clinical setting, understanding the roles of AMPK in cancer progression is essential. AMPK activation may not be beneficial in some cases.

## MATERIALS AND METHODS

### Patients and tissue samples

For the earlier RPPA study, we examined frozen tissue lysates for 139 protein markers from 101 patients with stages I-IV lung cancer who had not received neoadjuvant or adjuvant therapy [12]. For our current study, we were able to analyze only 55 of these 101 frozen tissue lysates (there was not enough protein for other 55 samples) for PKR expression by Western blotting. Immunohistochemical staining for PKR and p-AMPK^T172^ were performed previously on tissue microarray (TMA) samples from patients with stages I-IV disease who underwent resection of their primary cancer at The University of Texas MD Anderson Cancer Center between 1997 and 2001 [10,17]. All frozen tissues and specimens were obtained from the Lung Cancer Specialized Program of Research Excellence Tissue Bank at MD Anderson Cancer Center under a protocol approved by the MD Anderson Institutional Review Board. A Research Laboratory Protocols (LAB10-0704 and LAB07-0854) approved by the Institutional Review Board of our institution, and all patients have provided signed consent forms. Detailed clinical and pathologic information, including demographic data, smoking history, pathologic stage, and overall survival data, were available for all patients. The gender and race distributions of patients with lung cancer treated at MD Anderson were similar to those of patients throughout Texas and the rest of the United States.

### Western blotting

For the current analysis, frozen tumor tissues were washed twice in cold phosphate-buffered saline (PBS). Approximately 20 mg of tissue from each fresh sample was homogenized in 0.5 mL ice-cold lysis buffer (1% nonylphenoxypolyethoxylethanol [NP-40], 50 mM HEPES [pH 7.4], 150 mM sodium chloride, 1.5 mM magnesium chloride, 100 mM sodium fluoride, 1 mM ethylene glycol tetraacetic acid, 1 mM sodium orthovanadate, 10% glycerol, and 10 mM sodium pyrophosphate [Roche Applied Science]), which also contained freshly added protease and phosphate inhibitor. The lysates were spun at 14,000 X g in a microcentrifuge at 4°C for 10 minutes, and the resulting supernatants were used as tissue extracts for Western blot analysis. For cell lines, at 72 hours after transfection, the cell extracts were prepared, and Western blotting was performed as previously described [10, 31].

We obtained antibodies PKR (K-17) and LKB1 (Ley37D/G6, sc-32245) from Santa Cruz Biotechnology. We obtained the following antibodies from Epitomics: PKR phospho (pT451, 1120-1), p-eIF2α (S51), TSC2 (1613-1), mTOR (1612-1), and CaMKK beta (S1758). We obtained the following antibodies from Cell Signaling Technology: AMPK (2532), phospho-AMPK α (Thr172) (40H9) (2535), p-TSC2 (Ser1387) (5584), acetyl-CoA carboxylase (C83B10) (3676), phospho-acetyl-CoA carboxylase (Ser79) (3661), phospho-mTOR (Ser2448) (5536), phospho-LKB1 (Ser428) (C67A3) (3482), TAK1 (4505), and phospho-TAK1 (Thr184/187) (90C7) (4508). A mouse anti–β-actin antibody, which was used as the control antibody, was obtained from Sigma-Aldrich. Immunoreactive bands were detected and quantified using a LI-COR Odyssey infrared imaging system (LI-COR Biosciences).

### Cell lines and adenovirus transfection

Human lung cancer cell lines H1299 (p53 null), A549 (p53 wild), and H322 (p53 mutant) were obtained from the American Type Culture Collection (Manassas, VA). All cells were maintained in a medium containing 10% fetal bovine serum, 10 mM glutamine, 100 units/mL penicillin, and 100 μg/mL streptomycin (Life Technologies, Inc.) in a 5% carbon dioxide atmosphere at 37°C. Adenoviral vectors were constructed according to a previously published method^10^. We previously developed an adenoviral vector carrying either the wild-type PKR gene or a mutant form (PKRΔ6). The PKRΔ6 products have a deletion of six amino acids (361-366) between catalytic domains IV and V and cannot autophosphorylate or activate substrates ^10^. The transduction efficiencies of adenoviral vectors in H1299, A549, and H322 cancer cell lines were determined by infecting cells with Ad-LacZ and then quantifying the titers needed to transduce at least 70% of the cells.

### ATP, glucose uptake, and lactate production assay

The cellular ATP, glucose uptake, and lactate production were measured in H1299 and A549 cancer cells after transfection for 72 hours with PBS (control), Ad-PKR, Ad-PKRΔ6, or Ad-Luc. The ATP assay uses the highly sensitive “firefly” reaction to determine the level of cellular ATP as an indirect measure to assess the number of viable cells. The ATP assay was used for luminometric measurement according to the manufacturer's standard protocol (Roche Applied Science). ATP was extracted from the cells by adding 50 μL of tumor cell extraction reagent to each well. After mixing thoroughly, we incubated the microplates for 20–30 minutes at room temperature before transfering 50 μL of medium from each well to a white plate. Then 50 μL of luciferin-luciferase counting reagent was added. The microplates were measured using a count integration time of 1 second in a luminometer (BioTek). The count is directly proportional to the ATP content and the cell number. To measure cellular glucose uptake, cells were cultured and transfected with PBS (control), Ad-PKR, Ad-PKRΔ6, or Ad-Luc. At the time of collection, the cells were washed twice with glucose-free medium with normal serum content. The cells were then incubated with glucose-free medium containing 100 μM 2-NBDG (Invitrogen) for 3 hours, washed three times with PBS, and detached for fluorescence-activated cell sorting (FACS) analysis. The mean fluorescence intensity of the cells was obtained using a Becton Dickinson FACSCalibur flow cytometer. Lactate levels in the medium were detected with a lactate analyzer (Accutrend) after 72 hours of adenoviral vector transfection. The procedure was performed according to the manufacturer's instructions.

### Inhibition of p-AMPK expression by compound C and siRNA

We used compound C, an inhibitor of AMPK, and siRNA to inhibit p-AMPK protein expression. Compound C was obtained from Sigma Chemical Company. AMPK and control siRNA was purchased from Santa Cruz Biotechnology. To determine whether compound C or siRNA inhibits AMPK, Ad-PKR was added 24 hours before compound C or siRNA treatment. The H1299 and A549 cells were seeded in 6-well plates (2×10^5^ cells/well) in RPMI1640 medium overnight; the next day we added Ad-PKR for 24 hours and then added 10 mM compound C, AMPK siRNA (5 μg), or control siRNA for an additional 48 hours. Cells were collected at the end of treatment for flow cytometry analysis and Western blotting.

### Flow cytometry analysis

Flow cytometry analysis has been described previously [10]. Briefly after each treatment, cells were harvested; pelleted by centrifugation; resuspended in PBS containing 50 μg/mL propidium iodide, 0.1% Triton X-100, and 0.1% sodium citrate; and vortexed prior to FACS analysis (Becton-Dickenson FACScan; FL-3 channel).

### Statistical analysis

We used continuous data instead of median cut off for PKR and RPPA markers for association analysis. We determined the protein-protein relationship using Fisher's exact test and Spearman's correlation coefficient. *In vitro* data reported in the figures represent the mean of three independent experiments with SD. *P* values less than 0.05 were considered significantly different from untreated or treated controls. PKR biomarker were assigned to either low- or high-level groups based on the median score (cutoff point for biomarkers). The Kaplan-Meier method was used to estimate overall survival probability as a function of time for the study patients. The log-rank test was used to measure between-group differences in patient overall survival time.
